# Epidemiological investigation on hand hygiene knowledge and behaviour: a cross-sectional study on gender disparity

**DOI:** 10.1186/s12889-019-6705-5

**Published:** 2019-04-11

**Authors:** Lorna K. P. Suen, Zoe Y. Y. So, Simon K. W. Yeung, Kiki Y. K. Lo, Simon C. Lam

**Affiliations:** Squina International Centre for Infection Control, School of Nursing, The Hong Kong Polytechnic University, Hung Hom, Hong Kong, SAR China

**Keywords:** Gender, Hand hygiene, Hand washing, Survey, Knowledge, Hand drying, Public, Washrooms, Cross sectional study

## Abstract

**Background:**

The hand hygiene (HH) behaviour of the general public and its effect on illnesses are issues of growing importance. Gender is associated with HH behaviour. HH efficiency is a combination of washing efficiency and hand drying, but information about the knowledge level and HH behaviour of the general public is relatively limited. The findings of this cross-sectional study can substantially contribute to the understanding on the knowledge gap and public behaviour towards HH, thereby providing information on gender-specific health promotion activities and campaigns to improve HH compliance.

**Methods:**

An epidemiological investigation by using a cross-sectional study design on the general public was conducted either via an online platform (SurveyMonkey) or paper-and-pen methods. The hand-washing and -drying questionnaire was used for data collection.

**Results:**

A total of 815 valid questionnaires were collected. Majority of the respondents can differentiate the diseases that can or cannot be transmitted with poor HH, but the HH knowledge of the respondents was relatively inadequate. The female respondents had a significantly better HH knowledge than male respondents. The multiple regression analysis results also indicated that females had a significantly higher knowledge score by 0.288 towards HH than males after adjusting for age and education level. Although the majority of the respondents indicated that they performed hand cleaning under different specific situations, they admitted only using water instead of washing their hands with soap. More males than females dried their hands on their own clothing, whereas more females dried their hands through air evaporation. The average time of using warm hand dryers was generally inadequate amongst the respondents.

**Conclusions:**

Being a female, middle-aged and having tertiary education level are protective factors to improve HH knowledge. Misconceptions related to the concepts associated with HH were noted amongst the public. Self-reported practice on hand drying methods indicated that additional education was needed. The findings of this study can provide information on gender-specific health promotion activities and creative campaigns to achieve sustained improvement in HH practices.

## Background

Effective hand hygiene (HH) is important in preventing disease transmission in the clinical setting and community. The handwashing behaviour of the general public and its effect on illnesses are issues of growing importance [1–4]. Whether people can practice HH properly is uncertain. Many people overlook the importance of HH when engaging in activities that require handwashing. For example, < 40% of petting zoo visitors wash their hands upon exiting animal contact areas [2]. Handwashing with soap (HWWS) is the most effective way of removing pathogens from hands and preventing the spread of infectious diseases [5–7]. However, in a cross-sectional comparative study conducted in Bangladesh, a gap between perception and practice of HWWS was identified. In the study, majority of the respondents (90%) have knowledge about the importance of performing HWWS before eating and after defaecation, but only 21% and 88% respondents reported to do so, respectively [4].

Certain sociodemographic factors are associated with HH compliance. In an observational study, people who lived in urban districts, with high educational level and sufficient knowledge on infectious diseases have a high handwashing compliance rate [8]. Women are more likely to wash their hands than men after controlling for washroom characteristics and clustering effects associated with social norms [9]. In an experimental study by using unobtrusive observation of human behaviour, the presence of other people in a restroom makes written messages placed in the room as a successful reminder of handwashing amongst males [10].

HH efficiency is a combination of washing efficiency and hand drying. Empirical evidence indicated that handwashing is approximately 85% effective in removing microorganisms on hands, and hand drying provides a further reduction in transient flora [7]. Inadequately dried hands are more likely to transmit microorganisms compared with completely dried ones [11]. In comparison with scientific evidence associated with HH compliance amongst healthcare professionals [12–14], information about knowledge level and HH behaviour of the general public is relatively limited. Many studies have evaluated that HH behaviour focus on handwashing compliance and ignore the importance of hand drying [1, 3, 4, 8]. In a qualitative evaluation by using a grounded theory approach to understand hand drying practices amongst the public in Kenya, hand drying on a clean towel was found as an uncommon practice amongst the participants. Most women tend to dry their hands on their waist clothes when performing household chores, whereas men dry their hands on their trousers or a handkerchief [15]. Therefore, gender differences on preferred hand drying methods and the compliance of proper hand drying should be explored.

### Aim of the study

This study aims to determine the knowledge level and HH behaviour of the general public and attempt to identify gender differences on this issue. Both handwashing and hand drying behaviour in terms of gender disparity were investigated. The findings of this cross-sectional study can substantially contribute to the understanding on the knowledge gap and public behaviour towards HH, thereby providing information to gender-specific health promotion activities and campaigns for the improvement of HH compliance.

## Methods

*Study design:* An epidemiological investigation by using a cross-sectional study design.

### Subjects and procedure

Respondents aged ≥18 years old and residents of Hong Kong were recruited via snowball sampling method. A platform called SurveyMonkey was used to facilitate data collection [16]. SurveyMonkey is an online survey application that can facilitate the distribution of questionnaires via email; smartphones by using applications, such as WeChat and WhatsApp; and social media platform, such as Facebook. This survey application allows participants to access the questionnaire easily, and it analyses and exports results after responses have been collected [12, 17]. It also guides respondents to complete all items before exiting, thereby minimising the frequency of missing items. Questionnaires were also distributed using paper-and-pen format to a small number of participants, such as elderly who are not smartphone users. The research team used both data collection method to ensure a good coverage of respondents from different sociodemographic backgrounds, including age groups, educational level and working status to increase the generalisability of the findings.

#### Instrument

The hand-washing and -drying questionnaire was constructed based on an intensive literature review on key issues related to handwashing and drying [1, 4, 8, 18–23]. This questionnaire consisted of three parts. Part 1 mainly collects the sociodemographic data and personal habits. Part 2 focuses on knowledge on HH (12 items) that requires true or false responses. These items were selected according to the common myths and fallacies of HH reported in the literature, such as the misconceptions that hands should be held under water while lathering with soap and the adequate time used for hands rubbing before rinsing. The score ranged from 0 to 12, with high score indicating considerable knowledge level on HH. Part 3 comprises items related to self-reported handwashing and drying practices, such as the most common handwashing methods under specific conditions, preferred hand drying methods and public washroom facilities. Twenty respondents took an average of 12 min to complete all items during the pilot trial via SurveyMonkey.

The questionnaire was sent to a panel of 11 experts to determine if the relevant contents were covered by the instrument. These experts, who specialise in infection control and/or public health, are based in Australia, Korea, Taiwan, Hong Kong, Singapore, Malaysia and Switzerland. A content validity index of 0.92 was achieved. Test–retest reliability was performed on 20 subjects after 2 weeks of interval. The value for intraclass correlation coefficient (single measure) of the knowledge level on HH (part 2) was 0.941 (95% confidence interval = 0.857 to 0.976, *p* < 0.001). The item-to-item agreements for HH practice (Part 3) between the two measurements were satisfactory (weighted kappa and kappa values = 0.40 to 1.00 for 88.9% items; > 80% agreement on response for the remaining 11.1%). However, only some items in the original questionnaire were reported here due to the scope of this paper.

#### Data analysis

Descriptive statistics for sociodemographic characteristics, knowledge level on HH, self-reported HH and hand-drying practices of the respondents were presented. The association between categorical variables was examined using *x*^*2*^ test or Fisher’s exact test, where appropriate. Independent *t*-test was used to compare the gender differences in the knowledge score on HH. Backward multiple regression was conducted to identify the most parsimonious combination of gender and other extraneous variables in predicting knowledge score. SPSS version 25.0 (IBM Corporation, USA) was used for all statistical analyses. All statistical tests were two-sided, with a significance level set of *p <* 0.05.

## Results

The study was conducted from February to August 2018. A total of 1002 questionnaires were collected either via SurveyMonkey (*n* = 945), self-administered paper-and-pen format (*n* = 25) or paper-and-pen format with assistance (*n* = 32). The completion rate of the questionnaire on the SurveyMonkey was 80.2%, and the estimated time to complete was 11 min. After excluding the respondents with significant missing items (i.e. more than half of the items were found incomplete), 758 respondents from SurveyMonkey were included. Respondents from two community centres for the elderly (completed with assistance) and one group of university students from a community college (self-administered) completed the paper-and-pen questionnaire. Thus, 815 respondents were included for the analyses.

### Sociodemographic characteristics of respondents

The respondents were fairly distributed according to age group (18–29, 30–49, 50–59, 60 or above). Over half of these respondents were married or had partners (52.3%, *n* = 426), full-time job (59.5%, *n* = 485) and good to excellent health condition (56.4%, *n* = 463). The majority of the respondents attained tertiary or above education level (72.8%, *n* = 593) and had no comorbid illnesses (79.0%, *n* = 644). Only approximately 20.0% received influenza vaccination over the past 12 months (20.9%, *n* = 170). The characteristics were comparable between genders, except that the number of female participants was higher than the male participants with age ranging from 40 to 49 years old, are widowed/separated/divorced, and have a habit of wearing ring(s), artificial/acrylic nails (*p* < 0.001) or bracelets (*p* < 0.001). Conversely, more males than females have a habit of wearing watch (*p* < 0.05) (Table 1).

**Table 1:** Socio-demographic characteristics of respondents by gender

	Total (*n* = 815)	Male (*n* = 245)	Female (*n* = 570)	Test statistics & *p*-value
Variables	*n* (%)	*n* (%)	*n* (%)	
Age group				
18–29	215 (26.4)	74 (30.2)	141 (24.7)	0.003★**
30–39	138 (16.9)	47 (19.2)	91 (16.0)	
40–49	160 (19.6)	29 (11.8)	131 (23.0)	
50–59	213 (26.1)	62 (25.3)	151 (26.5)	
60 or above	89 (10.9)	33 (13.5)	56 (9.8)	
Marital status				
Single	332 (40.7)	101 (41.2)	231 (40.5)	0.021★*
Married/Partnered	426 (52.3)	136 (55.5)	290 (50.9)	
Widowed/Separated/Divorced	57 (7.0)	8 (3.3)	49 (8.6)	
Educational level				
Primary or below	34 (4.2)	9 (3.7)	25 (4.4)	0.838★
Secondary	188 (23.1)	59 (24.1)	129 (22.6)	
Tertiary /College or above	593 (72.8)	177 (72.2)	416 (73.0)	
Working status				
Full time	485 (59.5)	154 (62.9)	331 (58.1)	0.038★*
Part time	49 (6.0)	7 (2.9)	42 (7.4)	
Nil✡	281 (34.5)	84 (34.3)	197 (34.5)	
Comorbid illness				
No	644 (79.0)	192 (77.4)	452 (79.7)	0.513★
Yes	171 (21.0)	56 (22.6)	115 (20.3)	
Self-rated health condition				
Excellent	85 (10.4)	33 (13.5)	52 (9.1)	0.212★
Good	378 (46.4)	106 (43.3)	272 (47.7)	
Average	282 (34.6)	84 (34.3)	198 (34.7)	
Fair	60 (7.4)	17 (6.9)	43 (7.5)	
Poor	10 (1.2)	5 (2.0)	5 (0.9)	
Receive influenza vaccination over the past 12 months				
No	645 (79.1)	197 (80.4)	448 (78.6)	0.574★
Yes	170 (20.9)	48 (19.6)	122 (21.4)	
Have a habit of wearing ring(s)				
No	635 (77.9)	201 (82.0)	434 (76.1)	0.066★
Yes	180 (22.1)	44 (18.0)	136 (23.9)	
Have a habit of wearing artificial/acrylic nails	775 (95.1)	243 (99.2)	532 (93.3)	0.000***
No	40 (4.9)	2 (0.8)	38 (6.7)	
Yes				
Have a habit of wearing watch				
No	355 (43.6)	93 (38.0)	262 (46.0)	0.038★*
Yes	460 (56.4)	152 (62.0)	308 (54.0)	
Have a habit of wearing bracelet				
No	698 (85.6)	234 (95.5)	464 (81.4)	0.000★***
Yes	117 (14.4)	11 (4.5)	106 (18.6)	
Format for completing the questionnaire				
Via ‘Survey Monkey’	758 (93.0)	229 (93.5)	529 (92.8)	0.808★
Paper and pen (self-administered)	25 (3.1)	6 (2.4)	19 (3.3)	
Paper and pen (with assistance)	32 (3.9)	10 (4.1)	22 (3.9)	

Fisher’s exact test

★ Chi-square test

* Statistically significant at *p* < 0.05; ** Statistically significant at *p* < 0.01; *** Statistically significant at *p* < 0.001

✡retired/unemployed/housewife/student/voluntary job

### Knowledge level towards HH

In general, the majority of the respondents can differentiate the diseases that can or cannot be transmitted with poor HH. With regards to the concepts related to HH, the knowledge level of the respondents was relatively poor. The majority of the respondents misunderstood that always keeping the hands clean may decrease the body’s defence mechanism (79.0%, *n* = 644), hands should be held under water while lathering (64.8%, *n* = 528), and an alcohol-based hand sanitiser containing 40% alcohol was sufficient for hand disinfection (56.4%, *n* = 460). Slightly more than half of the respondents correctly answered the items related to the statement that lathering the hands for 10 s before rinsing is insufficient for hand disinfection (57.1%, *n* = 465). Over 30% of the respondents believed that the temperature of water may make a difference to the cleaning effect of hand cleaning (31.3%, *n* = 255). In general, female respondents had a significantly better knowledge level towards HH than males (9.38 ± 1.75 vs 9.06 ± 1.73; *p* < 0.05) (Table 2).

**Table 2:** Knowledge level on hand hygiene by gender (*n* = 815)

		Total (*n* = 815)	Male (*n* = 245)	Female (*n* = 570)	Test statistics & *p*-value
		*n* (%)	*n* (%)	*n* (%)	
Which of the following diseases can be transmitted by poor hand hygiene?					
1	Diarrheal disease				
	True ϕ	739 (90.7)	217 (88.6)	522 (91.6)	0.189★
	False	76 (9.3)	28 (11.4)	48 (8.4)	
2	URTI or ILI				
	True ϕ	700 (85.9)	205 (83.7)	495 (86.8)	0.272★
	False	115 (14.1)	40 (16.3)	75 (13.2)	
3	Hand-foot-mouth				
	True ϕ	779 (95.6)	234 (95.5)	545 (95.6)	1.000★
	False	36 (4.4)	11 (4.5)	25 (4.4)	
4	HIV				
	True	187 (22.9)	45 (18.4)	142 (24.9)	0.046★*
	False ϕ	628 (77.1)	200 (81.6)	428 (75.1)	
5	Skin ulcer				
	True ϕ	605 (74.2)	185 (75.5)	420 (73.3)	0.601★
	False	210 (25.8)	60 (24.5)	150 (26.3)	
6	Eye infections				
	True ϕ	785 (96.3)	238 (97.1)	547 (96.0)	0.431★
	False	30 (3.7)	7 (2.9)	23 (4.0)	
7	Diabetes				
	True	27 (3.3)	2 (0.8)	25 (4.4)	0.009**
	False ϕ	788 (96.7)	243 (99.2)	545 (95.6)	
Are the following statements correct?					
8	Always keeping your hands clean may lower our body defense mechanism				
	True	644 (79.0)	203 (82.9)	441 (77.4)	0.091★
	False ϕ	171 (21.0)	42 (17.1)	129 (22.6)	
9	Hands should be held under water while lathering with soap				
	True	528 (64.8)	119 (48.6)	168 (29.5)	0.000★***
	False ϕ	287 (35.2)	126 (51.4)	402 (70.5)	
10	An alcohol-based hand sanitizer that contain 40% alcohol is sufficient for hands disinfectant				
	True	460 (56.4)	132 (53.9)	223 (39.1)	0.000★***
	False ϕ	355 (43.6)	113 (46.1)	347 (60.9)	
	(Answer should be 60%)				
11	Rubbing my hands until soap forms a lather for 10 s before rinsing is enough for hand disinfection				
	True	350 (42.9)	148 (60.4)	317 (55.6.0)	0.217★
	False ϕ	465 (57.1)	97 (39.6)	253 (44.4)	
	(Answer should be 20 s)				
12	Temperature of water makes no difference in terms of the cleansing effect of hand cleaning				
	True ϕ	560 (68.7)	159 (64.9)	401 (70.4)	0.138★
	False	255 (31.3)	86 (35.1)	169 (29.6)	
	Total number of correct: (0 to 12)	9.28 ± 1.75	9.06 ± 1.73	9.38 ± 1.75	0.017▲*
	mean ± standard deviation				

ϕ correct answer

Fisher’s exact test

★ Chi-square test

▲ Independent *t-*test

* Statistically significant at *p* < 0.05; ** Statistically significant at *p* < 0.01; *** Statistically significant at *p* < 0.001

### Multiple regression analyses

Backward multiple regression was conducted to identify the most parsimonious combination of gender (male as referent), age group (18–29 as referent age, 30–49, 50–59, 60 or above), marital status (single as referent, married/partnered, widowed/separated/divorced), education level (primary or below as referent, secondary, tertiary or above), comorbid illnesses and working status (full-time as referent, part-time, retired/housewife/unemployed/voluntary) in predicting the knowledge score. The assumptions of linearity, data multicollinearity, homoscedasticity and distribution of residuals and the absence of influential cases were checked and met. After conducting backward regression, the model that included gender, age group (30–49 and ≥ 60 years old) and two dummy variables of education level (secondary and tertiary levels or above) was the most parsimonious combination (F [5, 809] = 27.78, *p* < 0.001, adjusted *R*^*2*^ = 0.141). The final model suggested that females had a significant higher knowledge score by 0.288 towards HH than males after adjusting for age (30–49 and ≥ 60 years old) and education level (secondary or tertiary level or above). After adjusting for other variables in the final model, the respondents aged 30–49 years old had a significant high knowledge score by 0.268, whereas respondents ≥aged 60 years old had a score reduction by 0.450 when compared with the reference group (18–29 years old). The respondents with secondary or tertiary level of education also had a significant increase in knowledge score by 1.825 and 2.482, respectively, compared with those with a low education level (primary or below, Table 3).

**Table 3:** Possible predictors on knowledge towards hand hygiene using regression analyses (final model)

Model	Variables entered	Unstandardized coefficients		*t*	Sig.	Adjusted R^2^	F value (df) (Sig.)
		Beta	Std error				
	(Constant)	6.806	0.342	19.919	0.000	.141	F(5, 809) = 27.781, *p* < 0.001***
	Gender	0.288	0.125	2.314	0.021		
	Age group (30–49)	0.268	0.123	2.183	0.029		
	Age group (60 or above)	−0.450	0.214	−2.108	0.035		
	Education level (Secondary)	1.825	0.330	5.525	0.000		
	Education level (Tertiary or above)	2.482	0.325	7.628	0.000		

Dependent variable: Total correct in knowledge questionnaire towards hand hygiene

Gender: Female (using male as the referent)

Age group (30–49), Age group (60 or above) (using age 18–29 as referent)

Education level (Secondary), Education level (Tertiary or above) (using primary school or below as referent)

### Self-reported hand cleaning and drying practices

The majority of the respondents indicated that they perform situational hand cleaning, including before and after handling food/cooking (> 98.0%), before eating (90.4%), after urination/defaecation (> 99.0%), after garbage bag disposal (97.1%), after sneezing/coughing (82.5%) or when the hands are visibly dirty (99.6%). If applicable, respondents performed hand cleaning after feeding a child (94.8%), caring for a sick person (99.1%), after daily work (88.5%), after touching livestock(s) (99.2%), after touching animal waste (100.0%) or after gardening (99.0%). When performing hand cleaning, significantly higher number of females than males performed HWWS than using water only (*p* < 0.001) in all critical moments. Over 70% of the respondents indicated that they use only ≤10 s for handwashing.

More female respondents than males tended to use paper towel to turn off a faucet or use water to splash the faucet before turning it off to avoid recontaminating the hands after washing. Over 70% of the respondents performed HH often during infectious disease outbreaks (76%). More male respondents than female ones ignored handwashing if they are in a hurry (*p* < 0.01), when nobody is in the washroom (*p* < 0.05) or when they only urinated (*p* < 0.001).

With regards to hand drying, more male respondents than female ones dry their hands on their own clothing (46.5% versus 37.7%, *p* < 0.01), whereas more female respondents dry their hands through air evaporation (70.4% versus 67.3%, *p* < 0.05). The respondents generally prefer using paper towels supplied by the washrooms than their own personal towels/handkerchief/tissues. The most often adopted methods for hand drying of the respondents were the use of paper towels (96.4%), followed by warm hand dryers (83.6%), jet hand dryer (78.5%) and cloth towel rolls (15.1%). The majority of the respondents shakes their hands to get rid of excess water before drying (87.5%) and limit the use of paper towels to two pieces (90.2%). Over half of the respondents rub their hands when using a warm hand dryer (51.1%). The average time for using warm hand dryers was generally inadequate amongst respondents, with over 60% of the respondents taking ≤10 s when using warm (60.9%) or jet hand dryer (64%) (Table 4).

**Table 4:** Self-reported hand cleaning and hand drying practice by gender (*n* = 815)

		Total (*n* = 815)	Male (*n* = 245)	Female (*n* = 570)	Test statistics & *p*-value
		*n* (%)※	*n* (%)※	*n* (%)※	
	Hand cleaning				
1.	Before handling food or cooking				
	No	14 (1.7)	7 (2.9%)	7 (1.2)	0.000***
	Water only	357 (44.1)	145(59.9%)	212 (37.3)	
	Water and soap	433 (53.5)	88 (36.4)	345 (60.7)	
	ABHR	4 (0.5)	1 (0.4)	3 (0.5)	
	Wet wipes	2 (0.2)	1 (0.4)	1 (0.2)	
	Not applicable	5	3	2	
2.	After handling food or cooking				
	No	16 (2.0)	8 (3.3)	8 (1.4)	0.000***
	Water only	242 (30.0)	104 (43.5)	138 (24.3)	
	Water and soap	547 (67.9)	126 (52.7)	421 (74.3)	
	ABHR	1 (0.1)	1 (0.4)	0 (0.0)	
	Not applicable	9	6	3	
3.	Before eating				
	No	78 (9.6)	30 (12.2)	48 (8.4)	0.000***
	Water only	330 (40.5)	127 (51.8)	203 (35.6)	
	Water and soap	380 (46.6)	83 (33.9)	297 (52.1)	
	ABHR	10 (1.2)	2 (0.8)	8 (1.4)	
	Wet wipes	17 (2.1)	3 (1.2)	14 (2.5)	
4.	After urination				
	No	2 (0.2)	1 (0.4)	1 (0.2)	0.000***
	Water only	431 (52.9)	171 (69.8)	260 (45.6)	
	Water and soap	382 (46.9)	73 (29.8)	309 (54.2)	
5.	After defaecation				
	No	1 (0.1)	1 (0.4)	0 (0.0)	0.001***
	Water only	129 (15.8)	54 (22.0)	75 (13.2)	
	Water and soap	683 (83.8)	189 (77.1)	494 (86.7)	
	ABHR	2 (0.2)	1 (0.4)	1 (0.2)	
6.	After feeding a child				
	No	30 (5.2)	12 (8.0)	18 (4.2)	0.009**
	Water only	253 (43.5)	72 (48.0)	181 (41.9)	
	Water and soap	288 (49.5)	60 (40.0)	228 (52.8)	
	ABHR	4 (0.7)	2 (1.3)	2 (0.5)	
	Wet wipes	7 (1.2)	4 (2.7)	3 (0.7)	
	Not applicable	233	95	138	
7.	After caring for a sick person				
	No	6 (0.9)	3 (1.5)	3 (0.6)	0.000***
	Water only	70 (10.0)	44 (21.8)	70 (10.0)	
	Water and soap	497 (10.9)	120 (59.4)	497 (70.9)	
	ABHR	127 (18.1)	35 (17.3)	127 (18.1)	
	Wet wipes	1 (0.1)	0 (0.0)	1 (0.1)	
	Not applicable	114	43	71	
8.	After daily work				
	No	90 (11.5)	32 (13.9)	58 (10.5)	0.219★
	Water only	248 (31.8)	98 (42.6)	150 (27.2)	
	Water and soap	410 (52.5)	96 (41.7)	314 (57.0)	
	ABHR	27 (3.5)	3 (1.3)	24 (4.4)	
	Wet wipes	6 (0.8)	1 (0.4)	5 (0.9)	
	Not applicable	34	15	19	
9.	When your hands are visibly dirty				
	No	3 (0.4)	2 (0.8)	1 (0.2)	0.000***
	Water only	22 (2.7)	10 (4.1)	12 (2.1)	
	Water and soap	79 (95.6)	231 (94.3)	548 (96.1)	
	ABHR	6 (0.7)	0 (0.0)	6 (1.1)	
	Wet wipes	5 (0.6)	2 (0.8)	3 (0.5)	
10.	After sneezing or coughing				
	No	143 (17.5)	58 (23.7)	85 (14.9)	0.000***
	Water only	215 (26.4)	90 (36.7)	125 (21.9)	
	Water and soap	330 (40.5)	79 (32.2)	251 (44.0)	
	ABHR	58 (7.1)	7 (2.9)	51 (8.9)	
	Wet wipes	69 (8.5)	11 (4.5)	58 (10.2)	
11.	After touching livestock(s)				
	No	4 (0.8)	0 (0.0)	4 (1.1)	0.000***
	Water only	39 (7.6)	19 (12.8)	20 (5.5)	
	Water and soap	421 (82.2)	116 (78.4)	305 (83.8)	
	ABHR	43 (8.4)	10 (6.8)	33 (9.1)	
	Wet wipes	5 (1.0)	3 (2.0)	2 (0.5)	
	Not applicable	303	97	206	
12.	After touching animal(s) waste				
	No	0 (0.0)	0 (0.0)	0 (0.0)	0.513
	Water only	15 (2.7)	6 (3.7)	9 (2.3)	
	Water and soap	498 (89.1)	141 (87.0)	357 (89.9)	
	ABHR	44 (7.9)	15 (9.3)	29 (7.3)	
	Wet wipes	2 (0.4)	0 (0.0)	2 (0.5)	
	Not applicable	256	83	173	
13.	After disposal of garbage bag				
	No	24 (2.9)	10 (4.1)	14 (2.5)	0.000***
	Water only	204 (25.0)	100 (40.8)	104 (18.2)	
	Water and soap	570 (69.9)	131 (53.5)	439 (77.0)	
	ABHR	15 (1.8)	4 (1.6)	11 (1.9)	
	Wet wipes	2 (0..2)	0 (0.0)	2 (0.4)	
14.	After gardening				
	No	6 (1.0)	2 (1.3)	4 (0.9)	0.000***
	Water only	165 (28.2)	69 (43.1)	96 (22.5)	
	Water and soap	411 (70.1)	89 (55.6)	322 (75.6)	
	ABHR	4 (0.7)	0 (0.0)	4 (0.9)	
	Not applicable	229	85	144	
15.	Would you perform hand hygiene more often during infectious disease outbreaks?				
	No	196 (24.0)	61 (24.9)	135 (23.7)	0.721★
	Yes	619 (76.0)	184 (75.1)	435 (76.3)	
16.	How much time do you usually lather your hands with soap before rinsing?				
	Less than 5 s	133 (16.3)	54 (22.0)	79 (13.9)	0.003★**
	5–10 s	476 (58.4)	142 (58.0)	334 (58.6)	
	11–19 s	102 (12.5)	30 (12.2)	72 (12.6)	
	20 s or more	104 (12.8)	19 (7.8)	85 (14.9)	
17.	Use a paper towel that you have used to dry your hands to turn off the faucet				
	Always	165(20.2)	41 (16.7)	124 (21.8)	0.003★**
	Sometimes	289 (35.5)	73 (29.8)	216 (37.9)	
	Never	361 (44.3)	131 (53.5)	230 (40.4)	
17.	Use a new paper towel to turn off the faucet				
	Always	46 (5.6)	21 (8.6)	25 (4.4)	0.039★*
	Sometimes	146 (18.0)	38 (15.5)	108 (18.9)	
	Never	623 (76.4)	186 (75.9)	437 (76.7)	
18.	Use water to splash the faucet before turning it off				
	Always	175 (21.5)	35 (14.3)	140 (24.6)	0.000★***
	Sometimes	346 (42.5)	91 (37.1)	255 (44.7)	
	Never	294 (36.0)	119 (48.6)	175 (30.7)	
19.	Ignore handwashing if in a hurry				
	Always/Sometimes	86 (10.6)	40 (16.3)	46 (8.1)	0.001★**
	Never	729 (89.4)	205 (83.7)	524 (91.9)	
20.	Ignore handwashing when nobody in the washroom				
	Always/Sometimes	49 (6.0)	22 (9.0)	27 (4.7)	0.024★*
	Never	766 (94.0)	233 (91.0)	543 (95.3)	
21.	Ignore handwashing if I have only urinated				
	Always/Sometimes	96 (11.8)	49 (20.0)	47 (8.2)	0.000★***
	Never	719 (88.2)	196 (80.0)	523 (91.8)	
	Hand drying				
22.	Rub hands on own clothing				
	Always	52 (6.4)	25 (10.2)	27 (4.7)	0.005★**
	Sometimes	277 (34.0)	89 (36.3)	188 (33.0)	
	Never	486 (59.6)	131 (53.5)	355 (62.3)	
23.	Air evaporation				
	Always	146 (17.9)	64 (26.1)	82 (14.4)	0.000★***
	Sometimes	420 (51.5)	101 (41.2)	319 (56.0)	
	Never	249 (30.6)	80 (32.7)	169 (29.6)	
24.	Use personal towel or handkerchief				
	Always	92 (11.3)	28 (11.4%)	64(11.2%)	0.242★
	Sometimes	218 (26.7)	56 (22.9%)	162 (28.4%)	
	Never	505 (62.0)	161 (65.7%)	344 (60.4%)	
25.	Use own disposable tissue				
	Always	320 (39.3%)	82 (33.5)	238 (41.8)	0.009★**
	Sometimes	360 (44.2%)	109 (44.5)	251 (44.0)	
	Never	135 (16.6%)	54 (22.0)	81 (14.2)	
26.	Paper towels supplied by the washroom				
	Always	592 (72.6)	172 (70.2)	420 (73.7)	0.590★
	Sometimes	194 (23.8)	64 (26.1)	130 (22.8)	
	Never	29 (3.6)	9 (3.7)	20 (3.5)	
27.	Warm hand dryer				
	Always	165 (20.2)	52 (21.2)	113 (19.8)	0.691★
	Sometimes	517 (63.4)	157 (64.1)	360 (63.2)	
	Never	133 (16.3)	36 (14.7)	97 (17.0)	
28.	Jet hand dryer				
	Always	152 (18.7)	48 (19.6)	104 (18.2)	0.544★
	Sometimes	487 (59.8)	150 (61.2)	337 (59.1)	
	Never	176 (21.6)	47 (19.2)	129 (22.6)	
29.	Cloth towel rolls				
	Always	17 (2.1)	5 (2.0)	12 (2.1)	0.282★
	Sometimes	106 (13.0)	39 (15.9)	67 (11.8)	
	Never	692 (84.9)	201 (82.0)	491 (86.1)	
30.	How many paper towels do you commonly used				
	One	365 (45.4)	106 (44.0)	259 (46.0)	0.324★
	Two	370 (46.0)	115 (47.7)	255 (45.3)	
	Three	57 (7.1)	14 (5.8)	43 (7.6)	
	Four or more	12 (1.5)	6 (2.5)	6 (1.1)	
	Not applicable	11	4	7	
31.	If warm hand dryer is used, how do you usually position your hands?				
	Rubbing the hands during drying	378 (51.1)	126 (56.8)	252 (48.6)	0.045★*
	Hold the hands stationary during drying	362 (48.9)	96 (43.2)	266 (51.4)	
	Not applicable	75	23	52	
32.	Average time for using warm hand dryer (in seconds)				
	Less than 5 s	56 (7.6)	22 (10.0)	34 (6.6)	0.188
	5–10 s	391 (53.3)	104 (47.0)	287 (56.0)	
	11–20 s	219 (29.9)	72 (32.6)	147 (28.7)	
	21–30 s	46 (6.3)	14 (6.3)	32 (6.3)	
	31–40 s	14 (1.9)	6 (2.7)	8 (1.6)	
	41 s or more	7 (1.0)	3 (1.4)	4 (0.8)	
	Not applicable	82	24	58	
33.	Average time for using jet hand dryer (in seconds)				
	Less than 5 s	75 (11.1)	24 (11.4)	51 (11.0)	0.692
	5–10 s	357 (52.9)	105 (49.8)	252 (54.4)	
	11–20 s	188 (27.9)	65 (30.8)	123 (26.5)	
	21–30 s	32 (4.7)	10 (4.7)	22 (4.7)	
	31–40 s	17 (2.5)	4 (1.9)	13 (2.8)	
	41 s or more	6 (0.9)	3 (1.4)	3 (0.6)	
	Not applicable	140	34	106	
34.	Shaking my hands to get rid of excess water before drying				
	Always	427 (52.4)	147 (60.0)	280 (49.1)	0.003★**
	Sometimes	289 (35.5)	80 (32.7)	209 (36.7)	
	Never	99 (12.1)	18 (7.3)	81 (14.2)	

Abbreviation: *ABHR* Alcohol based hand rub

Fisher’s exact test

★ Chi-square test

*Statistically significant at *p* < 0.05

**Statistically significant at *p* < 0.01

***Statistically significant at *p* < 0.001

※ Not applicable cases were excluded from percentage calculation and the analyses

## Discussion

In general, the majority of the respondents can differentiate the diseases associated with poor HH, with female respondents having a better overall knowledge on HH than males (9.38 vs 9.06 out of 12). The misconceptions of the respondents related to HH were identified. For example, the majority of the respondents misunderstood that always keeping the hands clean may lower the body’s defence mechanism, hands should be held under water while lathering, water temperature may induce a difference in the cleaning effects of hand cleaning and lathering for 10 s before rinsing is enough for hand disinfection. In fact, the time taken for handwashing and degree of friction generated during lathering are more important than water temperature in removing dirt and microorganism, and warm water causes skin irritations and is not environmentally friendly [7, 24, 25].

The inadequacy in the knowledge level is also in accordance with the self-reported practices of the respondents. Only 12.8% of the respondents indicated that they generally lather their hands with soap for ≥20 s before rinsing, with the percentages in female respondents slightly higher than those in males (14.9% vs 7.8%). This finding agreed with an observational study conducted in Ghana, wherein the majority of the participants perform handwashing for < 5 s [9]. The results of the multiple regression analyses also indicated that females had a significantly higher knowledge score by 0.288 towards HH than males after adjusting for age and education level. The age of 30–49 years old and high educational level were the protective factors for improved HH knowledge.

Although the majority of the respondents indicated that they perform hand cleaning under different specific situations, such as before and after cooking, before eating, after urination or defaecation, after disposal of garbage bag and after sneezing/coughing; they admitted that they only use water or, in rare occasions, use alcohol-based hand rub (ABHR) or wet wipes for hand cleaning instead of performing HWWS. Empirical evidence suggested that HWWS reduces the incidence of diarrhoeal diseases, respiratory infections and influenza and has been ranked the most cost-effective intervention for disease control by breaking the chain of transmission [3, 5, 6, 9, 10]. According to predictions, significantly higher number of female respondents than male ones expressed that they performed HWWS than using water only (*p* < 0.001) in all critical moments. Nearly half of the respondents believed that 40% alcohol is sufficient for hand disinfection by using ABHR. However, ABHR does not kill all types of germs, such as norovirus, *Clostridium difficile* and some parasites; thus, it is not recommended for use when the hands are visibly dirty or greasy, such as after gardening or doing outdoor activities [24].

Significantly higher number of males than female ones ignored handwashing if they are in a hurry, when nobody is in the washroom or when they only urinated. Aunger [6] also reported that factors such as business, tiredness or hunger can discourage male respondents from performing HH behaviour in their study. HH behaviour may be affected by others in the washrooms at the same time due to clustering effect [9]. HH is considered a social norm that is an effective driver to follow other’s behaviour in a relevant social group [5, 6]. Similarly, females’ high compliance can be also associated with their tendency to practice socially acceptable behaviour [26].

Female respondents have a habit of wearing ring(s), artificial/acrylic nails or bracelets. Conversely, more males than females have a habit of wearing a watch. The bacterial load was 2.63-fold higher on ringed hands than on nonringed hands significantly [27, 28]. Wearing rings other than a wedding ring, a bracelet or a watch and having long nails were associated with poor HH amongst hospital workers [29]. Therefore, the public should be reminded to pay special attention to these regions during handwashing.

Over 70% of the respondents perform HH often during infectious disease outbreaks. The association between HH behaviour and H1N1 or SARS pandemics suggested that public education campaigns are effective in altering HH behaviour during the peak periods of outbreak occurrences [30].

With regards to hand drying, more males than females dry their hands on their own clothing, whereas more females dry their hands through air evaporation than males. Drying hands on dirty clothes can compromise the benefits of handwashing [15]. Hands that are inadequately dried are more likely to transmit microorganisms when compared with those that have been completely dried [11]. Over half of the respondents rub their hands when using a warm hand dryer. The manner by which users place their hands under the hand dryer may also affect the number of remaining bacteria on hands. Yamamoto et al. [31] used a contact-plate method to evaluate the effects of hot air dryers when the hands are rubbed together or stationary. The rubbing process may tend to draw out commensal bacteria to the skin’s surface from deep inside the pores and under the fingernails. However, additional scientific evidence should be gathered to verify this causal relationship.

The most often adopted methods for hand drying of the respondents were the use of paper towels, followed by warm hand dryers, jet hand dryers and cloth towel rolls. Similar to the results of previous studies, paper towel is the most common hand-drying method [5, 32]. Paper towels can effectively dry hands, remove bacteria and cause less contamination in washrooms [33]. However, the use of paper towels can have adverse effects on waste disposal and environmental sustainability [34]. By contrast, conventional hand dryers have less environmental impact than paper towels [34]. However, conventional hand dryers are much slower than paper towels or jet hand dryers, taking approximately 45 s to eliminate only 3% of residual water [11]. The average time for using warm hand dryers was generally inadequate amongst respondents, with the majority of the respondents using ≤10 s when using warm or jet hand dryers. As a result, a significant amount of water remaining on the hands may easily recontaminate the hands after touching the surface environment, such as door handles upon leaving washrooms. More female respondents than males tend to use paper towels to turn off a faucet or use water to splash the faucet before turning it off to avoid recontaminating the hands after washing. However, this practice can increase the use of water and paper towels [24]. Hands-free faucet with motion sensor, doors with automatic control or even washrooms without doors were recommended.

The findings of this cross-sectional study contributed to the understanding on the knowledge gap and public behaviour towards HH and the gender differences towards this issue. This information can inform gender-specific health promotion activities and creative campaigns to improve HH compliance and achieve sustained improvement in HH practices.

### Limitations

This study has some limitations. HH is a socially desirable and morally laden behaviour. Therefore, the respondents may over-report the situation. Future studies that will adopt the nonobtrusive monitoring of HH behaviour may be conducted to provide an unbiased evaluation of actual behaviour. The network of the research team used convenience sampling. A relatively high percentage of the respondents attained tertiary education or above which may have an effect on HH behaviour. Quota sampling that takes into account age distribution and socioeconomic status may be considered in future surveys for a representative sample. Sanitation and facility in washrooms are the major factors affecting people’s handwashing practices [4, 35]. Future studies should be carried out to understand how washroom facilities can induce HH behaviour. HH knowledge and practices may also vary according to ethnicity and its associated washroom facilities. A wide-scale survey that compares the knowledge and HH behaviour of people residing in underdeveloped, developing and developed countries should be conducted to understand this topic from an international perspective.

## Conclusion

The study results showed that female respondents generally have a better knowledge level and more favourable HH behaviour than male ones. Being a female, middle-aged and having tertiary education level were the protective factors for improved HH knowledge. Misconceptions related to the concepts that are associated with HH were noted amongst the public. The most often adopted method for hand drying of the respondents was the use of paper towels. Self-reported practice on hand drying methods indicated that additional education was needed. The findings of this epidemiological investigation can provide information to gender-specific health promotion activities and creative campaigns to achieve sustained improvement in HH practices.

## Abbreviations

ABHR: alcohol-based hand rub
HH: hand hygiene
HWWS: handwashing with soap
